# OCT angiography metrics predict intradialytic hypotension episodes in chronic hemodialysis patients: a pilot, prospective study

**DOI:** 10.1038/s41598-021-86609-0

**Published:** 2021-03-30

**Authors:** Giuseppe Coppolino, Adriano Carnevali, Valentina Gatti, Caterina Battaglia, Giorgio Randazzo, Irma Figlia, Gemma Patella, Giorgio Fuiano, Michele Andreucci, Giuseppe Giannaccare, Vincenzo Scorcia, Davide Bolignano

**Affiliations:** 1 Renal Unit, University “Magna Graecia” of Catanzaro, Catanzaro, Italy; 2 Department of Ophthalmology, University Magna Graecia of Catanzaro, Viale Europa, Loc. Germaneto, Catanzaro, Italy

**Keywords:** Nephrology, Renal replacement therapy

## Abstract

In chronic hemodialysis (HD) patients, intradialytic hypotension (IDH) is a complication that increases mortality risk. We run a pilot study to analyzing possible relationships between optical coherence tomography angiography (OCT-A) metrics and IDH with the aim of evaluating if OCT-A could represent a useful tool to stratify the hypotensive risk in dialysis patients. A total of 35 eyes (35 patients) were analyzed. OCT-A was performed before and after a single dialysis session. We performed OCT-A 3 × 3 mm and 6 × 6 mm scanning area focused on the fovea centralis. Patients were then followed up to 30 days (10 HD sessions) and a total of 73 IDHs were recorded, with 12 patients (60%) experiencing at least one IDH. Different OCT-A parameters were reduced after dialysis: central choroid thickness (CCT), 6 × 6 mm foveal whole vessel density (VD) of superficial capillary plexus (SPC) and 6 × 6 mm foveal VD of deep capillary plexus (DCP). At logistic regression analysis, IDH was positively associated with baseline foveal VD of SCP and DCP, while an inverse association was found with the choroid. In Kaplan–Meier analyses of patients categorized according to the ROC-derived optimal thresholds, CCT, the 3 × 3 foveal VD of SCP, the 3 × 3 mm and 6 × 6 mm foveal VD of DCP and the 6 × 6 mm foveal VD of SCP were strongly associated with a higher risk of IDH over the 30-days follow-up. In HD patients, a single OCT-A measurement may represent a non-invasive, rapid tool to evaluate the compliance of vascular bed to HD stress and to stratify the risk of IDH in the short term.

## Introduction

Intradialytic hypotension (IDH) is a frequent complication of hemodialysis (HD). It’s associated to a high hospitalization rate and to end-organ damage, which is likely mediated through organ ischemia, and consequently to an increased mortality risk. IDH was estimated to affect one third of patients with a higher incidence in subjects with older age, diabetes and cardiovascular comorbidities, longer dialysis vintage, worst nutritional status or higher body mass index^1–5^. The pathogenesis of this condition remains partly unexplained although dysfunctions of the nervous autonomous system^6–8^ and various factors related to the hemodialytic procedure like rapid or excessive ultrafiltration^9^, excessive reduction in osmolality^10^ and reaction to the dialyzer membrane or machine tubing, seem to play a key role. Normal response to hypotension implicates various auto-regulatory mechanisms including the stimulation of sympathetic nervous system aiming at preserving blood flow in vital organs. In other districts, protection mechanisms intervene to maintain adequate blood flow to the metabolic demand of the tissues. This phenomenon can be observed, for instance, in renal arterioles, in coronary circulation or in retinal microcirculation^11–13^. The observation of ocular microcirculation gives us an exceptional chance to directly evaluate in vivo the reactions of human circulation to stress stimuli. Indeed, the ocular microcirculation is involved in systemic disease and early changes in vascular structures may predict the development of systemic vascular disorders^14–16^. Although the study of fundus oculi is mostly employed to assess hypertensive organ damage^16,17^, there is now evidence that also systemic hypotension may trigger important vascular changes and even retinal damage as the consequence of local hypoperfusion^18,19^. The effects of hemodialysis on choroid and retinal thickness have already been investigated by structural optical coherence tomography (OCT)^20^ and with the optical coherence tomography angiography (OCT-A)^21,22^, a new method for visualizing the retinal vasculature and choroidal vascular network that permits a tridimensional evaluation of vascular features such as vessels density. However, to date, no study focused on the possible clinical impact of frequent hypotensive episodes, as those occurring during hemodialysis, on the retinal structures.

In this pilot prospective study, we thus aimed at analyzing possible relationships between various OCT-A metrics and intradialytic hypotension and to evaluate whether OCT-A could represent a useful tool to stratify the hypotensive risk in dialysis patients.

## Methods

### Patients selection

Chronic hemodialysis patients from the Dialysis Unit of the University Magna Graecia of Catanzaro, Italy were screened for eligibility to participate into the study. The study was conducted in agreement with the Declaration of Helsinki for research involving human subjects and was approved by the local institutional review board (Comitato Etico Area Centro, Regione Calabria No 38 of 2018). A fully informed consent was obtained from all participants. All subjects were on regular renal replacement treatment with a rhythm of 4-h sessions/three times a week, had a stable dry-weight for at least 3 months before entering the study and had achieved a normotensive edema-free state. Exclusion criteria were dialysis vintage < 6 months, recent history of hospitalization for cardiovascular diseases, symptomatic pre-dialysis hypotension and severe cognitive or physical impairment^3,22^. Blood pressure (BP) was monitored using an automated sphygmomanometer integrated in the dialysis machine, every 20 min during and immediately following the dialysis session. Pre-dialysis blood pressure was measured in the non-access arm at the beginning of HD after 10-min rest with the patient seated in the dialysis chair before placement of a dialysis needle. Post-dialysis blood pressure was measured at the end of the session, 10-min after disconnecting the patient from the dialysis circuit. HD modalities were kept constant during the whole study period^23^. All the sessions consisted of standard HD using standard dialysis solutions, with bicarbonate buffer. The dialyzer used was a Flexya dialysis monitor (Bellco, Mirandola, Italy). Adequacy of dialysis was assessed using KT/V, calculated as the natural logarithm of the ratio between initial and final urea concentration. Dialysate sodium concentration was the same for all the patients at 140 meq/L and dialysate temperature maintained at 36.5 °C. Mean UF rate for hour never exceeded 0.6 mL/Kg/hour with the clinical endpoint to reach an adequate dry weight.

### OCT-A metrics

Measurement of OCT-A metrics was performed immediately before and after the first hemodialysis session of the week (Monday or Tuesday) at the Medical Retina & Imaging Unit of the Department of Ophthalmology, University Magna Graecia of Catanzaro. All measurements were performed by qualified ophthalmologists (A.C. and V.G) experts in retinal imaging, next to the dialysis unit.

Each patient underwent dilated fundus ophthalmoscopy, structural OCT and OCT-A. Ocular exclusion criteria included any other retinal diseases (including retinal vascular diseases, vitreoretinal diseases, history of central serous retinopathy, or macular dystrophies), ocular media opacity, any previous eye surgical intervention or laser photocoagulation in the study eye, poor quality images with significant artefact, inaccurate or incorrect segmentation at the level of the superficial capillary plexus (SCP) and deep capillary plexus (DCP), or subject’s inability to abstain from blinking or movement during image acquisition.

### OCT and OCT-A image acquisition

OCT-A was performed using XR Avanti AngioVue OCTA (Optovue, Fremont, California, USA). This system uses a split-spectrum amplitude decorrelation angiography (SSADA) algorithm and operates at 70,000 A-scans per second using a light source of 840 nm. SSADA detects variations in reflected OCT signal amplitudes between two consecutive scans^24^. Decorrelation is a mathematical function that quantifies this variation. SSADA splits the OCT signal into different spectral bands, thus increasing the number of usable image frames, in which each undergoes a decorrelation analysis^24^. Blood flowing through vessels causes a change in reflectance over time and results in localized areas of flow decorrelation between frames.

**Figure 1:**
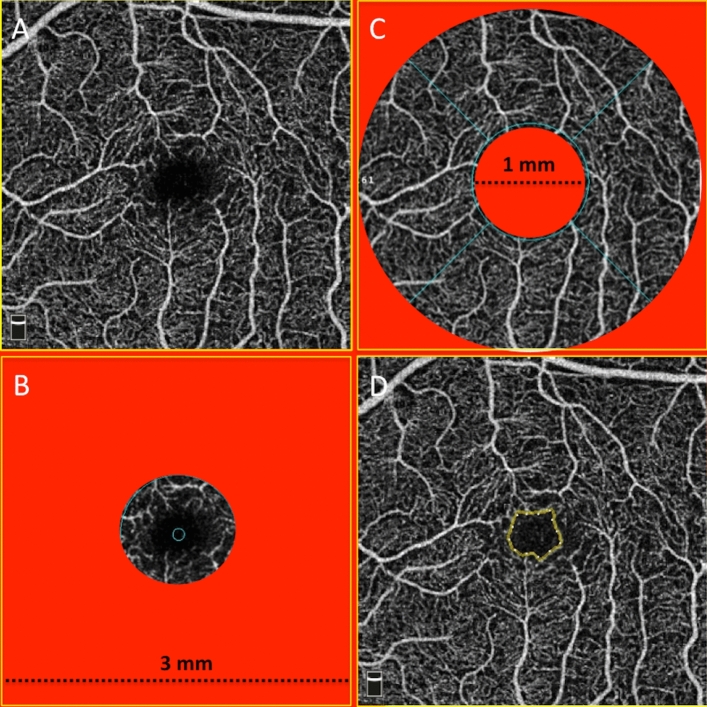
Schematic of optical coherence tomography angiography (OCTA) metrics. (**A**) OCTA image of whole SCP. (**B**) OCTA image of foveal region of the SCP delineated at ring of 1 mm. (**C**) OCTA image of parafoveal region of the SCP delineated between rings of 1 mm and 3 mm, ((**D**) OCTA image of full retina with the FAZ outlined in yellow.

En face images were acquired focusing the fovea centralis (Fig. 1).

Each scan consisted of 304 × 304 A-scans with two consecutive B-scans at each fixed position. To reduce motion artifacts, each scan consisted of one orthogonal horizontal and vertical scan^24^. We performed OCT-A 3 × 3 mm and 6 × 6 mm scanning area focused on the fovea centralis. The instrument software automatically segmented OCT-A scans into four en-face slabs: the SCP, the DCP, the outer retinal plexus and the choriocapillaris plexus. In this study we focus on SCP, DCP plexuses. The software detected the perfused vessel structures within an inner offset at − 3 to − 15 μm from the inner limiting membrane (ILM) for the evaluation of SCP and within an inner offset at − 15 to − 70 μm from the ILM for the DCP. The instrument software calculated automatically vessel density (VD) of SCP and DCP in the whole image, foveal and parafoveal zone and it also calculated automatically the foveal avascular zone (FAZ) area expressed in mm^2^ in the Retina plexus. VD was defined as the percentage of blood flow signal within a defined area. Foveal zone VD was defined as the area with a diameter of 1 mm; parafoveal zone VD was defined as the area with a diameter of 3 mm. For each patient were collected the following parameters: SCP VD and DCP VD of whole image, foveal and parafoveal zone and FAZ for both OCT-A 3 × 3 mm and 6 × 6 mm scans. Structural OCT was performed using RTVue OCT (Optovue Inc., Fremont, CA, USA), a high-speed and high-resolution spectral domain OCT device with central wavelength of 840 nm, scan rate of 26,000 A-scans/s, and axial resolution of 5 μm. In this study we were used horinzontal B-scan images centered on the fovea to manually measure central choroidal thickness (CCT) at the fovea: the vertical distance between the hyperscattering retinal pigment epithelium layer and the chorioscleral interface was measured manually using a software caliper built into the custom-made OCT image viewer. The three-dimensional macular scan protocol set to 7 × 7 mm containing 101 horizontal line scans each consisting of 513 A scans were used to calculated automatically central retinal thickness (CRT). All measurements were replicated in both eyes of the same patient and, if successful, a weighted mean of the two values was computed for each parameter and used for analysis.

### Prospective follow-up

After the baseline assessment, patients were prospectively followed up to 30 days and any hypotensive episode occurred during the correspondent ten dialysis sessions was recorded. Intradialytic hypotension episodes were defined as systolic blood pressure drop during dialysis greater than 20 mmHg or a decrease in mean arterial pressure of 10 mm Hg during dialysis with symptoms such as dizziness, headache, confusion, nausea, sweating requiring nurse intervention considering always a nadir systolic pressure < 90 mmHg^25^.

### Statistical analyses

The analyses were performed using the SPSS package (version 24.0; IBM corporation), the MedCalc Statistical Software (version 14.8.1) and the GraphPad prism software (version 8.4.2, San Diego, California USA).

Data were presented as mean ± SD for normally distributed values (at Kolmogorov–Smirnov test), median [IQ range] for variables with skewed distribution or frequency percentage. Differences between groups were determined by the unpaired T-test for normally distributed values, the Mann–Whitney U test for non-parametric values and the chi-square followed by a Fisher’s exact test for frequency distributions. Pre-post dialysis variations of retinal parameters were analyzed by a paired-T-test or by a Wilcoxon signed-rank test for non-parametric values.

Bivariate logistic regression analyses were performed to establish significant predictors of intradialytic hypotension among retinal parameters^26^. In addition, the Pearson (R) correlation was used to test correlations between such parameters and the absolute number of intradialytic hypotension episodes in each patient. A Receiver Operating Characteristics (ROC) analysis was employed to calculate the area under the curve (AUC) for every retinal parameter significantly associated with the outcome at bivariate logistic regression. AUCs were compared by a non-parametric approach. The best cut-off values were computed by the Youden index. Kaplan–Meier curves considering the time to the first intradialytic episode were generated for patients with retinal parameters above or below the optimal, ROC-derived threshold and compared by a Log-Rank test^27,28^. All results were considered significant if the p value was ≤ 0.05.

## Results

### Study cohort and baseline assessment

The source population consisted of 35 chronic haemodialysis patients. After the initial screening, 15 patients were excluded because refused to participate or due to the presence of significant retinal or ocular alterations hampering the reliability of measurements in both eyes. The final study cohort included 20 prevalent patients with a total of 35 eyes being correctly analyzed. The overall intra-patient concordance of measurements between the two eyes was very high for all parameters (R ranging from 0.899 to 0.995). Mean age of patients was 63.7 ± 11.4 years and the majority of them were male (69.2%). The median dialysis vintage was 25 months (IQR 12–41). Only 20% of patients were diabetics and the prevalence of cardiovascular comorbidities was relatively low (4.6–33%). Conversely, almost all patients (89.7%) were on anti-hypertensive treatment. Table 1 summarizes the main cohort data.

**Table 1:** Main laboratory and clinical characteristics of the whole study population and differences between subgroups.

	All N = 20	Hypotension N = 12	No-hypotension N = 8	*p*
**Age (years)**	63.7 ± 11.4	68.2 ± 11.3	57.4 ± 8.4	0.02
Gender (%Male)	69.2	65.5	62.5	0.44
Dry weight (kg)	66.2 ± 14.3	63.8 ± 11.2	69.7 ± 17.6	0.21
Kt/V	1.37 ± 0.15	1.4 ± 0.14	1.3 ± 0.12	0.18
Dialysis vintage (mo.)	25 [12–41]	25 [13–36]	25 [10–40]	0.81
Diabetes (%)	20.5	22.5	17.5	0.55
Past smokers (%)	30.3	29.3	30.1	0.57
History of myocardial ischemia (%)	33.3	35	25	0.64
Peripheral vasculopathy (%)	10.3	10.5	9.3	0.50
Cerebrovascular disease (%)	5.1	4.6	6.8	0.70
Hypertension (%)	89.7	75.4	100	0.08
Systolic blood pressure (mmHg)	136.4 ± 8.0	139.8 ± 6.1	143.1 ± 5.1	0.10
Diastolic blood pressure (mmHg)	71.5 ± 6.8	69.6 ± 7.3	74.4 ± 4.9	0.06
Serum phosphate (mg/dL)	5.8 ± 0.77	5.7 ± 0.9	5.9 ± 0.5	0.51
Serum calcium (mg/dL)	8.6 ± 0.73	8.6 ± 0.5	8.8 ± 0.9	0.39
Parathormone (pg/mL)	318 [245–476]	366 [285–576]	298 [199–386]	0.17
Albumin (g/dL)	4.3 ± 0.9	4.4 ± 0.6	4.1 ± 0.4	0.65
**LDL cholesterol (mg)/dL)**	96.2 ± 31.3	88.1 ± 29.1	107.7 ± 31.7	0.05
Total cholesterol (mg/dL)	222.4 ± 31.6	225.0 ± 31.1	218.7 ± 32.9	0.55
Triglycerides (mg/dL)	149.9 ± 48.0	144.8 ± 44.9	157.2 ± 52.7	0.43
Hematocrit (%)	34.5 ± 3.6	34.2 ± 2.7	34.8 ± 4.7	0.63
Hemoglobin (g/dL)	10.8 ± 0.9	10.7 ± 0.77	10.8 ± 1.17	0.94
White blood cells (n × 10^3^)	6.6 ± 1.5	6.4 ± 1.6	6.9 ± 1.4	0.24
Uric acid (mg/dL)	5.9 ± 1.0	5.9 ± 0.76	6.1 ± 1.4	0.59
C-reactive protein (mg/L)	3.7 [3.2–5.7]	3.9 [2.5–6.7]	2.7 [1.2–6.4]	0.35
**Ferritin (mg/dL)**	306 [219–370]	378 [306–927]	232 [151–289]	0.01
Serum iron (mg/dL)	72.6 ± 36.8	77.1 ± 40.6	66.2 ± 30.6	0.37
b2-microglobulin (mg/L)	26.2 ± .6.2	25.9 ± 7.1	26.6 ± 4.7	0.76
Urea (mg/dL)	142.9 ± 31.6	143.2 ± 34.6	142.5 ± 27.7	0.94
Fibrinogen (mg/dL)	331.4 ± 97.1	353.4 ± 86.8	299.6 ± 104.8	0.08
**Ocular parameters**				
CRT	276.7 ± 25.9	296.3 ± 118	251.8 ± 23.1	0.14
Choroid central thickness	300.8 ± 73.9	280.4 ± 72.6	330.1 ± 65.3	0.04
FAZ-SCP 3 × 3 mm	0.24 [0.16–0.34]	0.22 [0.16–0.26]	0.24 [0.16–0.39]	0.28
FAZ-SCP 6 × 6 mm	0.24 [0.15–0.30]	0.21 [0.15–0.26]	0.27 [0.18–0.39]	0.03
WHOLE-SCP 3 × 3 mm	41.5 ± 3	41.5 ± 4.4	42.3 ± 4	0.56
WHOLE-SCP 6 × 6 mm	47.3 ± 3.7	46.3 ± 5	47.3 ± 4.6	0.54
WHOLE-DCP 3 × 3 mm	47.8 ± 4.3	46.9 ± 5.4	47.8 ± 4.5	0.61
WHOLE-DCP 6 × 6 mm	44.2 ± 7	44.5 ± 5.8	44.6 ± 5.9	0.96
PARAFOVEA-SCP 3 × 3 mm	43 ± 7.5	44 ± 4.6	45.4 ± 4.1	0.34
PARAFOVEA-SCP 6 × 6 mm	48.1 ± 3.9	47.2 ± 4.6	48.8 ± 4.7	0.32
PARAFOVEA-DCP 3 × 3 mm	49.7 ± 4.7	48.4 ± 5.8	50.2 ± 4.7	0.32
PARAFOVEA-DCP6 × 6 mm	50.1 ± 6.7	50.1 ± 4.3	50.4 ± 7.2	0.86
FOVEA-SCP 3 × 3 mm	20.7 ± 12	19.9 ± 3.3	16 ± 7.9	0.04
FOVEA-SCP 6 × 6 mm	23.8 ± 9.3	24.7 ± 8.2	15.6 ± 8.1	0.002
FOVEA-DCP 3 × 3 mm	35.8 ± 8.9	37.1 ± 5.8	31.4 ± 8.1	0.02
FOVEA-DCP 6 × 6 mm	39.4 ± 11	41.2 ± 9.9	34.8 ± 9.7	0.04

*CRT* central retinal thickness, *FAZ* foveal avascular zone, *DCP* deep capillary plexus, *SCP* superficial capillary plexus.

### Effects of a single hemodialysis session on OCT-A metrics

**Figure 2:**
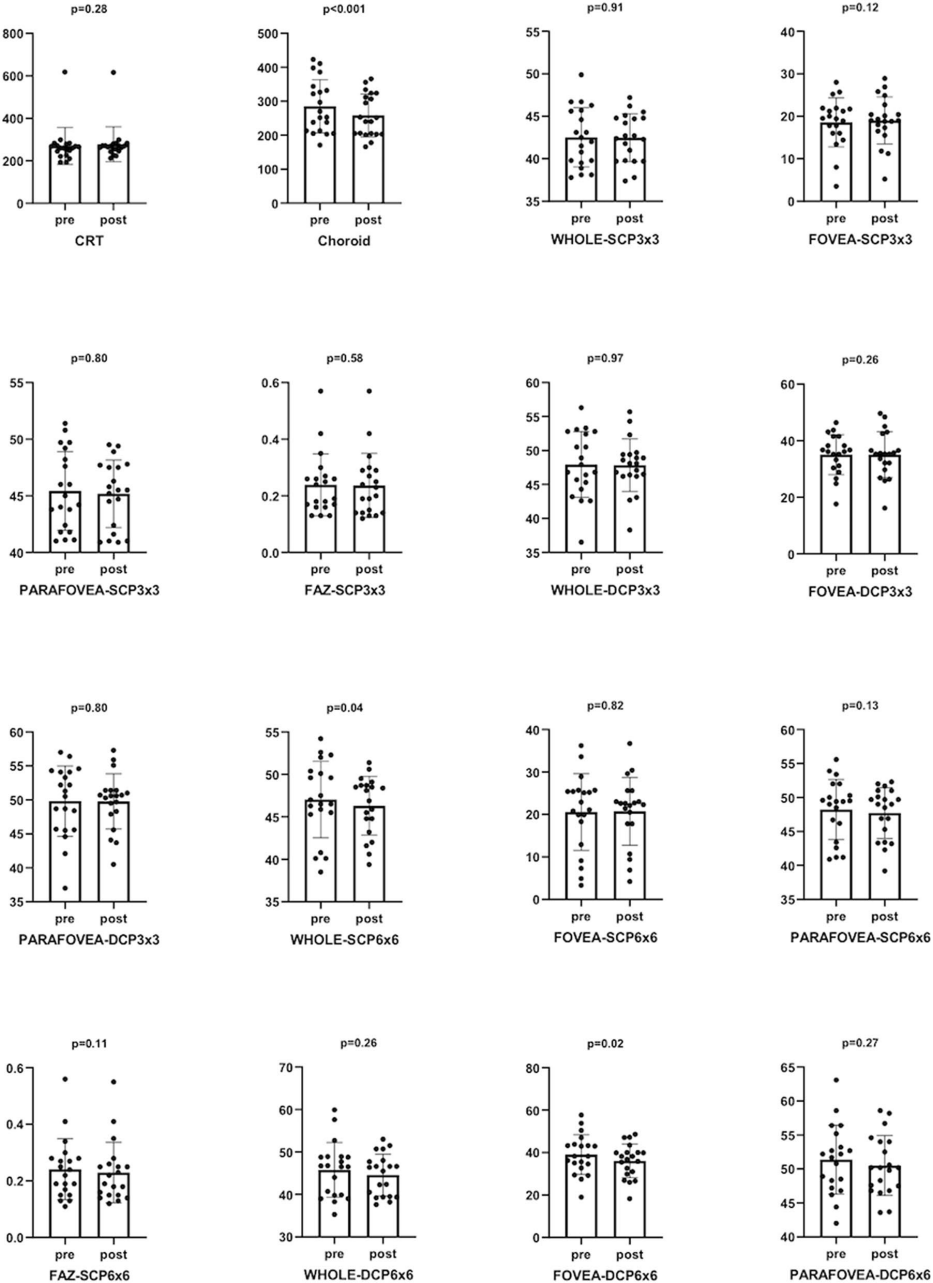
Pre-post dialysis variations in OCT-A metrics. A more prominent significant drop was found in choroidal parameters (Choroid p < 0.001, WHOLE-SCP6X6 and FOVEA-DCP6X6 p < 0.05) respect to retinal parameters.

All OCT-A metrics were measured before and after a single hemodialysis session. Overall, the majority of parameters remained unchanged at dialysis end, a significant reduction being noticed only for central choroid thickness (median Δ value 25, IQR 10–57; p < 0.001), 6 × 6 whole VD of SCP (median Δ value 1.4, IQR 0.6–3.5; p = 0.04) and 6 × 6 foveal VD of DCP (median Δ value 2.5, IQR 0.4–4.6; p = 0.02) (Fig. 2). Stratified subgroups analyses demonstrated a more prominent reduction in CCT (p = 0.03), as well as in 3 × 3 whole VD of SCP and DCP (p = 0.05 for both comparisons) in individuals not experiencing intradialytic hypotensive episodes as compared to others (Table 2).

**Table 2:** Pre-post dialysis change (Δ) in OCT-A metrics in the whole cohort and in subgroups.

	All N = 20	Hypotension N = 12	No-hypotension N = 8	*p*
ΔCRT	0 [− 3 to 2]	− 2 [− 3 to 2]	1 [− 1.7 to 2]	0.21
ΔChoroid central thickness	25 [10 to 57]	**20 [2** to **45]**	**38 [21** **to** **69.7]**	**0.03**
ΔFAZ-SCP 3 × 3 mm	0 [− 0.01 to 0.01]	0 [− 0.1 to 0.1]	0.01 [− 0.04 to 0.01]	0.17
ΔFAZ-SCP 6 × 6 mm	0.05 [− 0.04 to 0.01]	0.03 [− 0.02 to 0.01]	0.07 [− 0.02 to 0.04]	0.16
ΔWHOLE-SCP 3 × 3 mm	0.3 [− 1.6 to 1.7]	**0.1 [**− **2.7** to **1.5]**	**1 [**− **0.6** **to** **2.4]**	**0.05**
ΔWHOLE-SCP 6 × 6 mm	1.4 [0.6 to 3.5]	1.7 [0.5 to 3.6]	1.2 [0.6 to 3.2]	0.84
ΔWHOLE-DCP 3 × 3 mm	− 0.1 [− 3.2 to 1.2]	− **1.3 [**− **3.3** to **1.1]**	**0.3 [**− **0.8** **to** **2.3]**	**0.05**
ΔWHOLE-DCP 6 × 6 mm	− 0.6 [− 2.6 to 3.5]	− 0.6 [− 3.5 to 3.5]	0.05 [− 2.3 to 6.6]	0.50
ΔPARAFOVEA-SCP 3 × 3 mm	0.5 [− 1.6 to 1.6]	0.5 [− 2.8 to 1.3]	1.05 [− 0.1 to 2.2]	0.17
ΔPARAFOVEA-SCP 6 × 6 mm	1 [− 0.8 to 3.3]	2.3 [− 2.9 to 4.4]	0.2 [− 0.6 to 2.6]	0.20
ΔPARAFOVEA-DCP 3 × 3 mm	0.3 [− 3 to 1.9]	− 1.9 [− 3 to 1.5]	0.9 [− 2.1 to 4.2]	0.17
ΔPARAFOVEA-DCP 6 × 6 mm	− 1.3 [− 3 to 4.7]	− 1.3 [− 1.8 to 4.7]	− 0.9 [− 4.8 to 5.6]	0.76
ΔFOVEA-SCP 3 × 3 mm	− 0.5 [− 1.7 to 1.5]	0.5 [− 2.8 to 1.3]	− 0.5 [− 2.6 to 1.1]	0.76
ΔFOVEA-SCP 6 × 6 mm	0.5 [− 1 to 2.6]	2.4 [− 0.5 to 3]	0.2 [− 0.6 to 2.6]	0.21
ΔFOVEA-DCP 3 × 3 mm	− 0.1 [− 2.5 to 1.4]	− 0.1 [− 3.5 to 1.7]	− 0.1 [− 2.2 to 1.4]	0.89
ΔFOVEA-DCP 6 × 6 mm	2.5 [0.4 to 4.6]	− 1.3 [− 1.5 to 2]	− 0.9 [− 4.8 to 5.6]	0.38

*CRT* central retinal thickness, *FAZ* foveal avascular zone, *DCP* deep capillary plexus, *SCP* superficial capillary plexus.

Statistically significant differences between subgroups are shown in bold.

### Prospective follow-up and intradialytic hypotension

During the 30-day follow-up, 73 hypotensive episodes equivalent to an incidence rate of 3.65 episodes/pts/month (95% CI 2.86–4.58) were registered. All hypotension events were resolved with appropriate therapeutic management and no clinical sequelae. Twelve patients (60%) experienced at least one hypotensive episode (mean 6.08 ± 2.89). At baseline, these individuals were older and had significantly lower systolic and diastolic blood pressure and LDL cholesterol but higher serum ferritin levels as compared with individuals without hypotensive episodes. With respect to retinal parameters, they displayed a statistically thinner choroid (p = 0.04) and a reduced 6 × 6 FAZ (p = 0.03) but a larger 3 × 3 and 6 × 6 foveal VD of SCP (p = 0.04 and 0.002, respectively), as well as a larger 3 × 3 and 6 × 6 foveal VD of DCP (p = 0.02 and 0.04, respectively). No statistical differences were noticed with respect to other parameters. Table 1 resumes the main characteristics of the two study subgroups.

### Associations between OCT-A metrics and IDH

**Figure 3:**
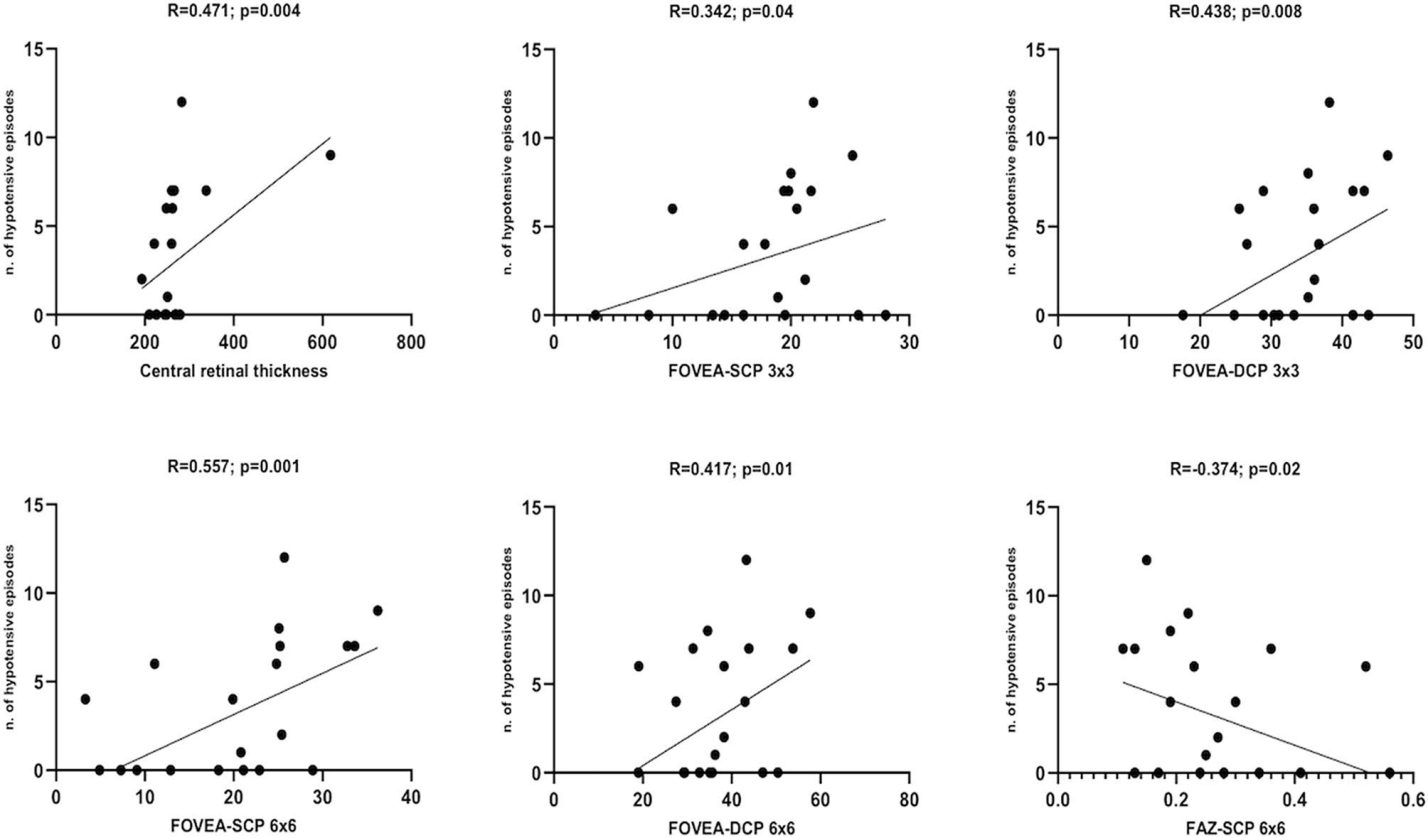
Correlations between number of hypotensive episodes and OCT-A parameters.

Pre-dialysis OCT-A metrics were tested separately into a bivariate logistic regression analysis to find possible associations with the occurrence of intradialytic hypotension. As summarized in Table 3, a statistically significant positive association was found with the 3 × 3 and 6 × 6 foveal VD of SCP (OR 1.122; 95% CI 1.001–1.276; p = 0.05 and OR 1.143; 95% CI 1.034–1.264; p = 0.009, respectively) and the 3 × 3 and 6 × 6 foveal VD of DCP (OR 1.130; 95% CI 1.011–1.263; p = 0.03 and OR 1.072; 95% CI 1.002–1.157; p = 0.04, respectively), while an inverse association was found with the CCTOR 0.990; 95% CI 0.979–0.999; p = 0.05). In addition, CRT and the 3 × 3 and 6 × 6 foveal VD of SCP and DCP were directly associated to the absolute number of hypotensive episodes (R spanning from 0.342 to 0.557), while the 6 × 6 FAZ was negatively correlated (R =  − 0.374, p = 0.02; Fig. 3, Table 4).

**Table 3:** OCT-A parameters significantly associated to the occurrence of intradialytic hypotension at logistic regression analyses.

Parameter	OR	95% CI	p
Choroid central thickness	0.990	0.979–0.999	0.05
FOVEA-SCP 3 × 3 mm	1.122	1.001–1.276	0.05
FOVEA-DCP 3 × 3 mm	1.130	1.011–1.263	0.03
FOVEA-SCP 6 × 6 mm	1.143	1.034–1.264	0.009
FOVEA-DCP 6 × 6 mm	1.072	1.002–1.157	0.04

*CRT* central retinal thickness, *FAZ* foveal avascular zone, *DCP* deep capillary plexus, *SCP* superficial capillary plexus.

**Table 4:** Significant associations (Pearson coefficient) between OCT-A parameters and the number of hypotensive episodes during follow-up.

Parameter	R	p
CRT	0.471	0.004
FOVEA-SCP 3 × 3 mm	0.342	0.04
FOVEA-DCP 3 × 3 mm	0.438	0.008
FOVEA-SCP 6 × 6 mm	0.557	0.001
FOVEA-DCP 6 × 6 mm	0.417	0.01
FAZ-SCP 6 × 6 mm	− 0.374	0.02

*CRT* central retinal thickness, *FAZ* foveal avascular zone, *DCP* deep capillary plexus, *SCP* superficial capillary plexus.

### Predictive value of OCT-A metrics on IDH

**Figure 4:**
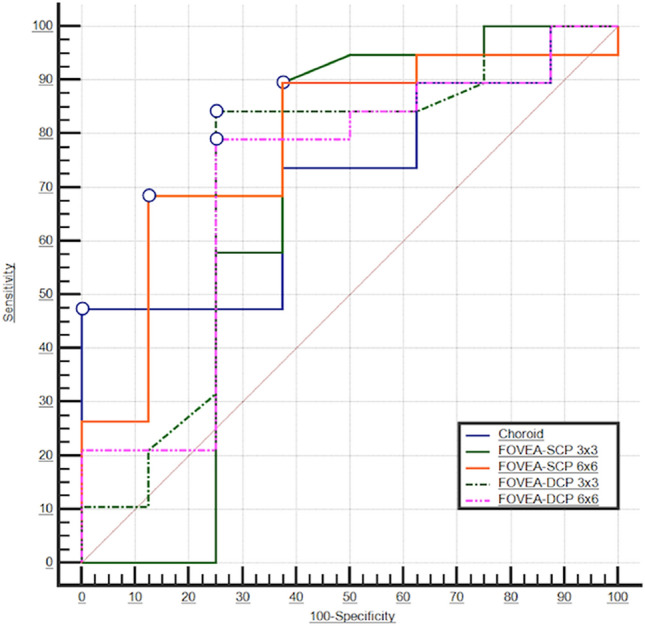
Areas under the curve (AUCs) of OCT-A parameters to identify HD patients experiencing hypotensive episodes. White circles indicate optimal thresholds (Youden index) for each variable. Differences in AUCs were statistically not significant.

The diagnostic capacity to identify patients with intradialytic hypotension episodes was tested by ROC analyses for each parameter significantly associated with the outcome at logistic regression. The AUC for 3 × 3 foveal VD of SCP was statistically not significant. Conversely, the AUCs of CCT, 3 × 3 foveal VD of DCP and 6 × 6 foveal VD of SCP and DCP were all significant (range 0.704 to 0.783; p ranging from 0.03 to 0.006), although not different from each other (Fig. 4). Table 5 provides a summary of ROC analyses of retinal parameters, including the best cut-off values computed for each variable able to discriminate patients with or without the outcome of interest.

**Table 5:** Areas under the curve (AUCs) and best cut-off values (Youden index) of OCT-A parameters to detect patients with intradialytic hypotensive episodes.

	AUC [95% CI]	p	Best cut-off	Sens.%	Spec.%
**Choroid central thickness**	**0.711 [0.535–0.886]**	**0.01**	** ≤ 242**	**47.3 [24.4–71.1]**	**100 [79.4–100]**
**FOVEA-DCP 3 × 3 mm**	**0.720 [0.532–0.908]**	**0.02**	** > 33.2**	**84.2 [60.4–96.6]**	**75 [47.6–92.7]**
**FOVEA-SCP 6 × 6 mm**	**0.783 [0.622–0.944]**	**0.006**	** > 22.9**	**68.4 [43.4–87.4]**	**87.5 [61.7–98.4]**
**FOVEA-DCP 6 × 6 mm**	**0.704 [0.516–0.892]**	**0.03**	** > 35.6**	**78.9 [54.4–93.9]**	**75 [47.6–92.7]**
FOVEA-SCP 3 × 3 mm	0.674 [0.460–0.889]	0.11	> 16	89.4 [66.9–98.7]	62.5 [35.4–84.8]

*FAZ* foveal avascular zone, *SCP* superficial capillary plexus.

Statistically significant AUCs are highlighted in bold.

**Figure 5:**
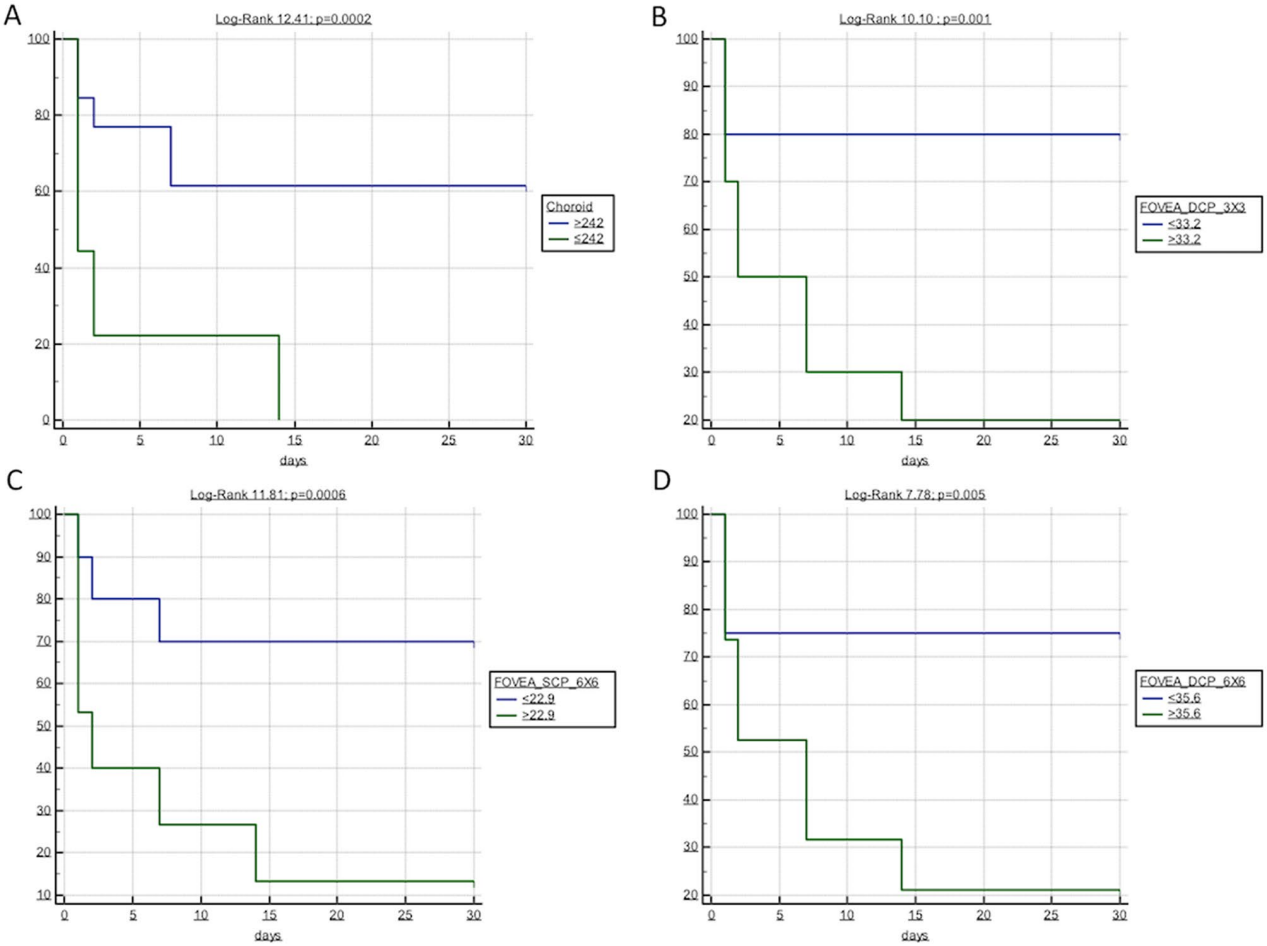
Kaplan–Meier survival curves of intradialytic hypertension-free patients according to the optimal ROC-derived cut-off for (**A**) Choroid, (**B**) 3 × 3 foveal DCP, (**C**) 6 × 6 foveal SCP and (**D**) 6 × 6 foveal DCP.

Kaplan–Meier curves were thus generated for patients categorized according to such ROC-derived optimal thresholds (Fig. 5). All the parameters tested in these analyses, namely the CCT, the 3 × 3 foveal VD of SCP, the 3 × 3 and 6 × 6 foveal VD of DCP and the 6 × 6 foveal VD of SCP, resulted strongly associated with a higher risk of intradialytic hypotension (Log-rank test ranging from 7.78 to 12.41) over the 30-days follow-up.

## Discussion

In our study, we demonstrated that chronic hemodialysis patients experiencing frequent IDH episodes showed a different ocular pattern with respect to those not experiencing this complication. Furthermore, a simple measurement of retinal and choroid parameters by OCT-A before a single dialysis session may help predicting the risk of following IDH in the short-term.

Currently, OCT-A and analytic softwares have facilitated automated and manual processing of macular perfusion data^29–31^. The effect of hemodialysis on perfused vessel density is still debated, with some studies reporting a significant reduction^21,32^ and some others indicating no differences^33^ or even a paradoxical increase^34^. In our study, we reported a barely or not significant drop of vessels dimension or density in choroidal and retinal analysis in the whole study population while, interestingly, a more prominent decrease in central choroid thickness and whole SCP and DCP were noticed between patients experiencing IDHs or not. Similar changes previously described in other studies employing OCT-A in HD patients were referred to a decrease in body weight, systolic blood pressure and serum osmolarity^35^. Blood pressure in patients on hemodialysis routinely exhibits marked variability, tending to be highest in the immediate pre-dialysis period and decreasing during the intradialytic period. The majority of patients experience an overall decline in BP, on average in the range of 30–40 mmHg, which was in line with the findings in our cohort. Such a typical trend of BP occurs without adverse symptoms, being well tolerated by patients. Occasionally hypotension could occur if fluid removal during hemodialysis exceeds the correct evaluated ultrafiltration rate. Patients in our analysis were stringently enrolled avoiding excessive or rapid fluid removal during observational period. Nevertheless, in line with common clinical practice in some subjects we recorded hypotensive events. OCT-A analysis in the IDH group with respect to patients not experiencing this complication showed a lower size in choroid at basal measurement and small changes after HD. Notoriously, the choroidal circulation has the highest blood supply per organ area and has a specific extrinsic autonomic regulation unlike retinal vessels^36^. Sympathetic innervation includes noradrenergic and neuropeptide fibers while parasympathetic innervation consists of cholinergic fibers^37,38^. Decrease in choroidal flow is mediated by sympathetic innervation through release of noradrenaline^6^ while an increase activates parasympathetic reflexes by nitric oxide release^34^. In dialysis patients, the particular response pattern of ocular vessels observed in the IDH group could reflect a more pronounced, overall dysautonomic state, a pathological condition that is a hallmark of uremia. Patients prone to IDH are known to have an amplified sensitivity to the Bezold–Jarisch reflex. This reflex begins when myocardial mechanoreceptors are activated in response to ventricular under filling and leads to vagal afferent inhibition of the medullary cardiovascular center. Consequently, the patient experiences a dramatic decrease in sympathetic nervous system activity with following arteriolar vasodilation, bradycardia, and IDH. This state is probably a consequence of uremic toxins interacting with the neural termination and affecting their activation through superoxide-dependent effects on neural activity in particular by reducing nitric oxide (NO) bioavailability and inducing systemic inflammation^39^. In IDH patients, the response to hypotension in single vascular districts aims at re-establishing a stable blood volume after fluid excess removal but these mechanisms are often inadequate. Interestingly, pre-dialysis choroid thickness showed an impressive ability to predict the occurrence of IDH, as showed by both diagnostic and survival analyses. Dysregulation of ocular blood flow was deeply studied and observed in diabetic retinopathy with significant neuronal and glial changes leading to a reduction in the functional hyperemia response and in basal blood flow^40^. Our findings could therefore be interpreted as a typical morphological adaptation of vascular bed in certain patients, suggesting that OCT-A could represent a surrogate, non-invasive way to evaluate autonomic vasomotor function in dialysis patients.

In our analysis, also VD of DCP and SCP plexuses in foveal shots predicted the occurrence of hypotensive episodes.

Differently from choroid, retinal circulation is similar to brain circulation but lacks of autonomic innervation. The capillary unit consists of a continuous endothelium and intramural pericytes. Structural retinal changes are known as early indicators of the presence and severity of coronary artery disease^37^, also predicting cardiovascular mortality^41,42^. Differences in retinal vessels density in the IDH group could also reflect endothelial dysfunction. In normal subjects the reduction of systemic blood pressure results in the constriction of pericytes and reduction in blood flow to choroidal and retinal capillaries mediated by oxidization and nitration stimuli^43–46^. Reduction in ocular blood pressure causes the release of vasodilator factors such as adenosine^47^ and N-methyl-D aspartic acid^48^.

In a healthy animal model, Tami et al.^49^ recently showed that a drop of ocular perfusion pressure under 80 mmHg, consequent to systemic hypotension, activates vasodilatory mechanisms able to maintain retinal blood flow. Feedback autoregulation was blunted by the administration of L-NAME (N omega-nitro-l-arginine methyl ester) a well-known inhibitor of nitric oxide synthase (NOS) used to show endothelium-dependent relaxations of arteries^50^. Maintenance of blood flow patency is age-related^51^. In this context, uremia was often considered a model of accelerated aging^52^ and serious vascular dysfunction. Exposure to the uremic toxic milieu and repetitive episodes of hypotension as observed in some HD patients could also lead to loss of this regulation system^53^.

Our study has some strengths and limits that deserve mentioning. The main strength was the homogeneity of cohort, the sequential pre-post dialysis evaluation of retinal parameters and a prospective phase that was long enough to capture a sufficient number of IDH episodes according to validated criteria. The main limit is probably represented by the small, single-centre evaluation and the lack of information on markers able to demonstrate dysregulation of autonomic nervous system. However, despite the limited number of cases analyzed, we reported a significant number of patients experiencing IDHs and a quite high absolute number of episodes over the follow-up period, which allowed to uncover significant associations between retinal parameters and hypotensive episodes at both logistic and survival analyses and to define a strong diagnostic capacity at ROC analyses for some of these parameters. In conclusion, in this pilot study, we found OCT-A as a potential tool to screen and measure the compliance of vascular bed to hemodialytic stress. Larger investigations are needed to confirm whether this approach may represent also an alternative way to stratify patients according to the risk of developing dangerous hypotensive complications in the short-mid-term.
